# MGnify: the microbiome sequence data analysis resource in 2023

**DOI:** 10.1093/nar/gkac1080

**Published:** 2022-12-07

**Authors:** Lorna Richardson, Ben Allen, Germana Baldi, Martin Beracochea, Maxwell L Bileschi, Tony Burdett, Josephine Burgin, Juan Caballero-Pérez, Guy Cochrane, Lucy J Colwell, Tom Curtis, Alejandra Escobar-Zepeda, Tatiana A Gurbich, Varsha Kale, Anton Korobeynikov, Shriya Raj, Alexander B Rogers, Ekaterina Sakharova, Santiago Sanchez, Darren J Wilkinson, Robert D Finn

**Affiliations:** European Molecular Biology Laboratory, European Bioinformatics Institute (EMBL-EBI), Wellcome Genome Campus, Hinxton, Cambridge, UK; School of Engineering, Newcastle University, Newcastle upon Tyne, UK; European Molecular Biology Laboratory, European Bioinformatics Institute (EMBL-EBI), Wellcome Genome Campus, Hinxton, Cambridge, UK; European Molecular Biology Laboratory, European Bioinformatics Institute (EMBL-EBI), Wellcome Genome Campus, Hinxton, Cambridge, UK; Google Research, Brain Team, Mountain View, CA, USA; European Molecular Biology Laboratory, European Bioinformatics Institute (EMBL-EBI), Wellcome Genome Campus, Hinxton, Cambridge, UK; European Molecular Biology Laboratory, European Bioinformatics Institute (EMBL-EBI), Wellcome Genome Campus, Hinxton, Cambridge, UK; European Molecular Biology Laboratory, European Bioinformatics Institute (EMBL-EBI), Wellcome Genome Campus, Hinxton, Cambridge, UK; European Molecular Biology Laboratory, European Bioinformatics Institute (EMBL-EBI), Wellcome Genome Campus, Hinxton, Cambridge, UK; Google Research, Brain Team, Mountain View, CA, USA; Department of Chemistry, University of Cambridge, Cambridge, UK; School of Engineering, Newcastle University, Newcastle upon Tyne, UK; European Molecular Biology Laboratory, European Bioinformatics Institute (EMBL-EBI), Wellcome Genome Campus, Hinxton, Cambridge, UK; European Molecular Biology Laboratory, European Bioinformatics Institute (EMBL-EBI), Wellcome Genome Campus, Hinxton, Cambridge, UK; European Molecular Biology Laboratory, European Bioinformatics Institute (EMBL-EBI), Wellcome Genome Campus, Hinxton, Cambridge, UK; Center for Algorithmic Biotechnology, St Petersburg State University, St Petersburg, Russia; European Molecular Biology Laboratory, European Bioinformatics Institute (EMBL-EBI), Wellcome Genome Campus, Hinxton, Cambridge, UK; European Molecular Biology Laboratory, European Bioinformatics Institute (EMBL-EBI), Wellcome Genome Campus, Hinxton, Cambridge, UK; European Molecular Biology Laboratory, European Bioinformatics Institute (EMBL-EBI), Wellcome Genome Campus, Hinxton, Cambridge, UK; European Molecular Biology Laboratory, European Bioinformatics Institute (EMBL-EBI), Wellcome Genome Campus, Hinxton, Cambridge, UK; Department of Mathematical Sciences, Durham University, Durham, UK; European Molecular Biology Laboratory, European Bioinformatics Institute (EMBL-EBI), Wellcome Genome Campus, Hinxton, Cambridge, UK

## Abstract

The MGnify platform (https://www.ebi.ac.uk/metagenomics) facilitates the assembly, analysis and archiving of microbiome-derived nucleic acid sequences. The platform provides access to taxonomic assignments and functional annotations for nearly half a million analyses covering metabarcoding, metatranscriptomic, and metagenomic datasets, which are derived from a wide range of different environments. Over the past 3 years, MGnify has not only grown in terms of the number of datasets contained but also increased the breadth of analyses provided, such as the analysis of long-read sequences. The MGnify protein database now exceeds 2.4 billion non-redundant sequences predicted from metagenomic assemblies. This collection is now organised into a relational database making it possible to understand the genomic context of the protein through navigation back to the source assembly and sample metadata, marking a major improvement. To extend beyond the functional annotations already provided in MGnify, we have applied deep learning-based annotation methods. The technology underlying MGnify's Application Programming Interface (API) and website has been upgraded, and we have enabled the ability to perform downstream analysis of the MGnify data through the introduction of a coupled Jupyter Lab environment.

## Graphical Abstract

**Graphical Abstract:**
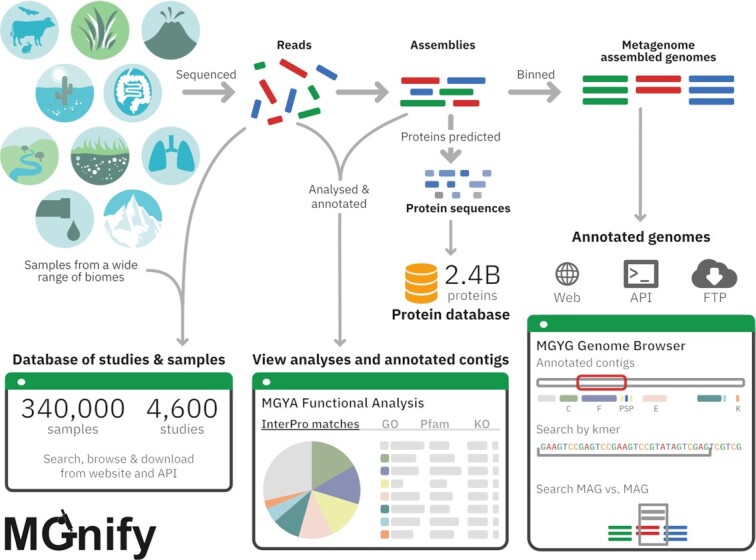
Overview of MGnify resources: the assembly and annotation of microbiome-derived sequences from a broad range of environments has given rise to new insights into microbial diversity and the functional repertoire they encode.

## INTRODUCTION

The number of investigations characterising microbial communities continues to grow at a rapid pace as increasingly diverse biomes (environments) are sampled and analysed in greater depth using modern nucleic acid sequencing technologies. This expansion represents various continually evolving methodologies ranging from DNA-based metabarcoding and metagenomic approaches to RNA-based metatranscriptomics, along with protein and metabolite profiling of communities through metaproteomics and metabolomics, respectively (1). MGnify is a centralised hub for the discovery of meta’omics sequence data and the provision of harmonised analysis, facilitating comparative analyses of datasets originating from different projects. MGnify's growth has mirrored the developments occurring in the wider research area. For instance, microbiome sampling is heavily skewed towards common biomes with a long tail of less frequently sampled environments. Currently, 297 different biomes are represented in the database with over half of MGnify's analyses originating from merely nine of them: human-faecal, -oral, -digestive system, -skin, and unspecified human; marine; soil; mammalian digestive systems; and mixed biome samples. However, as the range of sampled biomes continues to expand, coverage has concomitantly increased, which is reflected by nine distinct new biomes hosted in MGnify previously absent at the time of our last update (2): human hindgut; aquatic hypersaline microbial mats; mammalian foregut; arthropoda hindgut and oral; lab enriched anaerobic media; rhizoplane soil; annelida digestive system; and composting wood. In parallel, 53 biomes have more than doubled in their analyses count.

MGnify employs standardised (versioned) analysis pipelines allowing results to be interpreted in context with other datasets. All tools and pipelines are open and freely available within public repositories (https://github.com/EBI-Metagenomics) and all workflows are formally described in Common Workflow Language (CWL, https://www.commonwl.org/ (3)) and are gradually being deposited in WorkflowHub (https://workflowhub.eu/projects/9 (4)) to support easy reuse within the research community. MGnify works closely with the European Nucleotide Archive (ENA), which archives sample metadata, sequence reads, and assemblies. Researchers can submit pre-publication data to the ENA and request the assembly and/or analysis of those data by MGnify with results subsequently provided within the user's own private area of MGnify. Users may also request the assembly and/or (re-)analysis of any relevant public dataset available in the International Nucleotide Sequence Database Collaboration (INSDC) initiative.

Beyond data growth, both the field of microbiome research and MGnify as a resource are expanding into a new era of microbial coverage. Approaches for the recovery of genomes from environmental samples first appeared in 2004 (5), with reference-free approaches being developed in the following decade (6). Since then there has been a paradigm shift, as a result of the now routine large-scale recovery of genomes from metagenomes (so called metagenome assembled genomes, MAGs) (7,8). This approach has been applied extensively in the most-sampled biome, namely the human gut, where the Unified Human Gastrointestinal Genome catalogue provided draft genomes for 4644 prokaryotic species of which 70% lacked cultured representative genomes (9).

Herein, we describe major recent updates to the MGnify resource aimed at streamlining access to the MGnify analyses and derived data products. These updates include improvements to the website and associated Application Programming Interface (API), the provision of enhanced analysis options directly from the web pages, and a substantial overhaul of the MGnify protein database combined with a new release comprising more than 2.4 billion non-redundant sequences. Together, these updates expand the utility of the MGnify resource by improving interconnections between data products, and enhancing access to both MGnify-generated results and user-defined downstream analyses.

### Expansion of data in MGnify

Since our last update (2), we have continued to expand the content of MGnify through a combination of user-requested analyses and analyses of targeted public datasets. Since we can achieve substantially richer functional annotations with assembled datasets compared to raw read analysis, our primary focus has been to provide assembly and analysis of metagenomic and metatranscriptomic datasets. In addition to the improved protein predictions from assembled sequences, they also allow us to provide higher-level annotations, such as pathway predictions (KEGG (10), Genome Properties (11)) and prediction of biosynthetic gene clusters (BGC) using antiSMASH (12) and our inhouse tool for BGC prediction (https://github.com/Finn-Lab/SanntiS). We remain committed in ensuring sequence data is appropriately archived as well as analysed, so all assembled public datasets are also submitted to the ENA as a linked third party annotation. The provision of assembly as a service by MGnify allows users without the sufficient compute resources to undertake this form of analysis, thus democratising the process of metagenomic assembly for the community. We work closely with the ENA and continually seek to improve the data flow between the two resources. Notably, we have developed a private brokering procedure that has streamlined the process of submitting private/pre-publication assemblies on behalf of the data owner into their own account. This significantly reduces effort on the part of the submitter who previously had to fetch the assemblies from a file sharing system, and then upload the data to the ENA themselves. The timescale for assembly of data (both public and private) is highly variable as it depends on factors such as the microbial diversity and sequencing depth of the sample, the number of concurrent requests, as well as the availability of shared compute capacity. As such, assemblies take weeks rather than days to produce and analyse via MGnify. However, we endeavour to keep users updated throughout the process of assembly and analysis.

**Figure 1.**
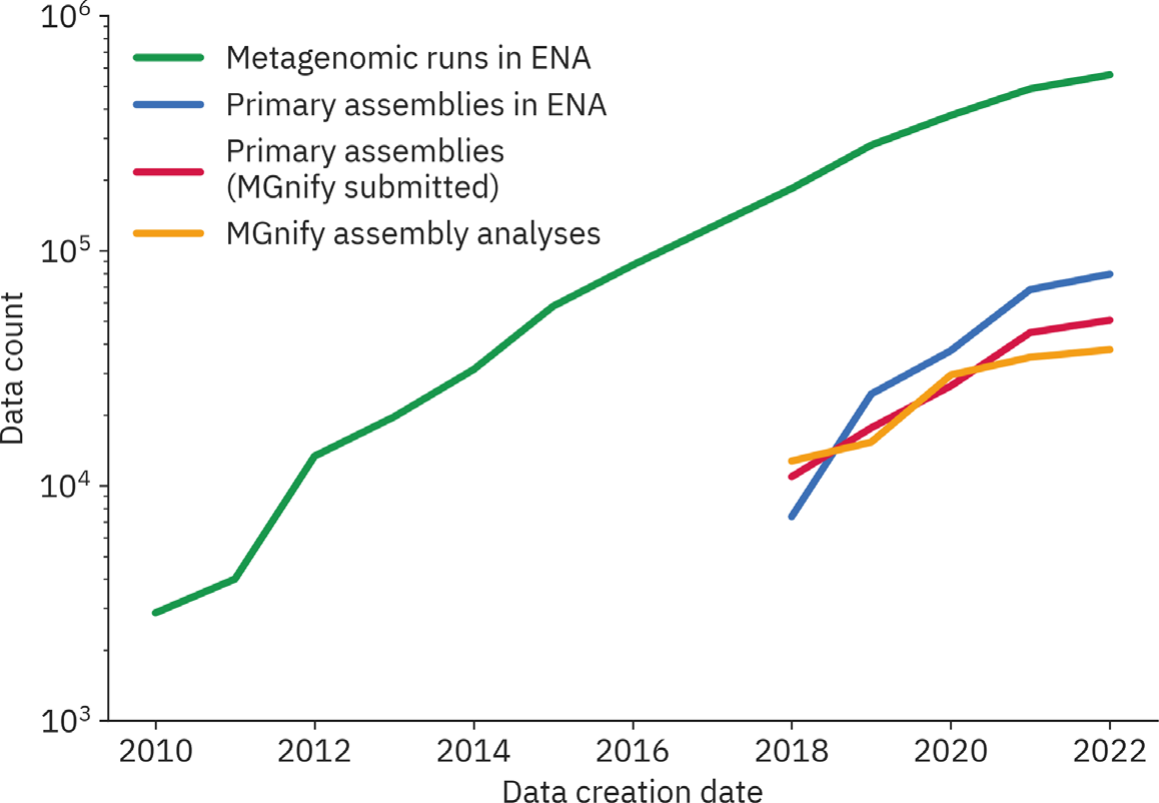
The number of assembled metagenomics datasets in the ENA and MGnify over time. MGnify launched assembly and analysis of assemblies in 2017, however counts of primary assemblies submitted to the ENA are only available from 2018 due to a change in recording. Until 1 August 2022, the MGnify team has generated and submitted an assembly for 88% of all primary assembled metagenomic datasets in the ENA.

The prioritisation of metagenomics assembly coupled with their corresponding submission as primary metagenomes to the ENA has resulted in a further 33K MGnify generated assemblies in the last three years (see Figure 1). In fact, the vast majority of assembled metagenomics raw reads in the ENA (44 758 out of 50 705, 88%) have an assembly generated by MGnify. While a substantial portion of raw metagenomic data currently available remains unassembled, several reasons explain why an associated assembly may not exist: (a) environmental samples (such as soil and aquatic) often represent particularly diverse environments and consequently, can be extremely memory intensive to assemble with standard algorithms; (b) there will be cases where the sequencing coverage of a particular sample is simply too low to allow successful assembly, further compounding the memory issues; (c) some samples are mislabelled and actually represent metabarcoding datasets.

Alongside the analysis of assembled datasets, we continue to provide analysis of amplicon (also termed metabarcoding) datasets, as these still represent a substantial portion of the available microbiome data. Currently, MGnify contains 382 093 amplicon analyses, which represent 10% of the total amplicon data available in the ENA. Overall MGnify contains 475 390 analyses that pertain to 343 695 distinct samples, arranged into 4601 studies.

### Support for long-read sequencing technologies

Although the vast majority of metagenomic data is still generated using short-read technologies (predominantly Illumina), long-read sequencing data from PacBio and Oxford Nanopore Technologies sequencing platforms has become increasingly available. Therefore, we have expanded our pipelines to better support the assembly and analysis of both long-read only datasets and hybrid datasets, i.e. where the same sample has been sequenced using both a long- and short-read approach. As with all the MGnify analysis workflows, the pipeline providing support for long-read sequencing technologies is formally described in CWL and available within the MGnify GitHub repository (https://github.com/EBI-Metagenomics/mgnify-lr). Users can request long-read or hybrid assembly of existing datasets via the same mechanism used for short-read analysis (i.e. by generating an analysis request from the MGnify website) but are prompted to explicitly highlight the relevant datasets required for hybrid assemblies.

### Enrichment of microbiome metadata

**Figure 2.**
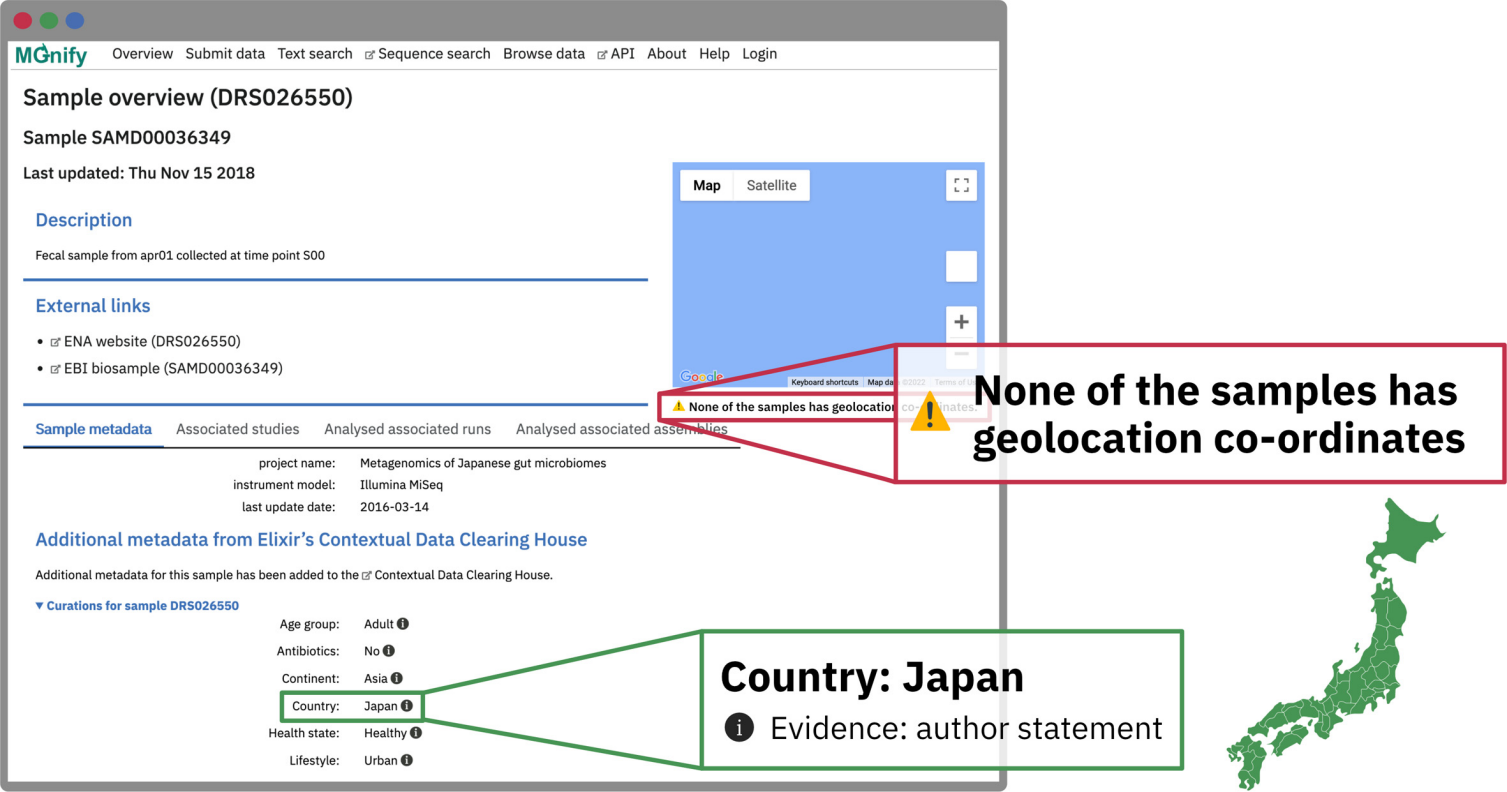
A sample in MGnify that lacks structured geolocation information in the ENA. However a Contextual Data Clearing House curation is available, listing the country of origin as Japan.

One of the major limiting factors in the interpretation of microbiome data and analysis is the availability of descriptive metadata. Comprehensive metadata describing the sample can be inconsistently submitted alongside the sequence record and therefore, crucial context for interpretation may be lacking. In many cases, additional metadata can be found associated with the sample in the free text of a publication. Recent advances in text mining have enabled the extraction of relevant metadata terms from the free text of publications and the deposition of those metadata into annotation databases. Nassar *et al.* (13) describe the extraction of metadata from 19 900 metagenomic studies present in MGnify. These annotations—describing metadata, such as geographic locations and sequencing methods—are now shown within the MGnify website, alongside the structured metadata associated with samples and studies in the ENA (see Figure 2). Of the 1746 publications in MGnify, 1398 have annotations extracted from publications. These are particularly useful when structured sample metadata is missing: for example, MGnify contains 1120 agricultural soil samples lacking location metadata, but 143 of these samples (13%) now show geographic annotations on the MGnify website through their linked publications.

We have supplemented this source of metadata with that from the Contextual Data Clearing House (CDCH, https://www.ebi.ac.uk/ena/clearinghouse/api/). The CDCH enables curation of sample metadata by correcting and adding records. A curation is a single attribute:value pair, which is associated with a sample, sequence, or study, and supported by an evidence assertion. For example, the sample DRS026550 is missing geolocation data in the ENA but a CDCH curation lists Country:Japan as evidenced by an author statement. Like the publication annotations, these CDCH curations are now shown alongside existing metadata from the ENA when viewing a sample in MGnify.

### Latest release of the MGnify protein database

The MGnify protein database is a resource comprising all protein sequences derived from the analyses of assembled data in MGnify. This resource has been used for multiple streams of ongoing research. Examples include: (i) the protein database was cited as a crucial source of additional sequences for multiple sequence alignments (MSAs) used by AlphaFold2 (14), with sequences from metagenomic sources enriching poorly represented protein families in more classical protein databases; (ii) Eiamthong *et al.* (15) successfully mined the protein database in search of novel polyethylene terephthalate (PET) hydrolases using sequence homology to a known PETase; (iii) Inoue *et al.* (16) used the sequence set to determine the relationship between specific clades of metabolically important Ni-containing carbon monoxide dehydrogenases (Ni-CODHs) and their biome distribution and (iv) Kazlauskas *et al.* (17) utilised the protein database in their analysis of the diversity and evolution of B-family DNA polymerases.

**Figure 3.**
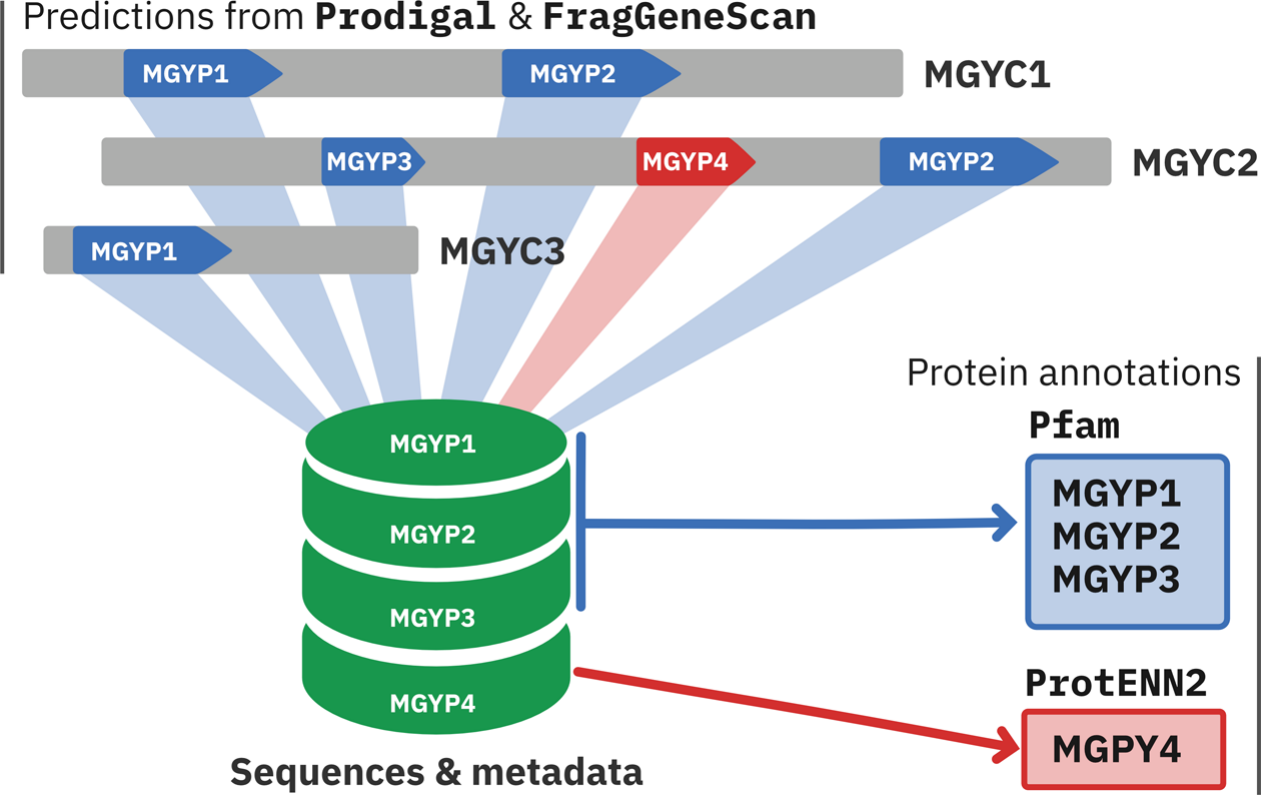
Schematic of the protein database. Proteins are predicted on each contig (MGYC) using Prodigal (18) and FragGeneScan (19). The sequence and metadata of unique proteins (MGYP) are stored in a MySQL database. Annotations from Pfam (23) and ProtENN2 (Bileschi *et al.*, in prep., (24)) for each protein are also stored.

Since its initial release in 2017, the MGnify protein sequence set has grown steadily over the years in line with the growth of MGnify assemblies. To overcome the challenges associated with the processes used to collate the sequence set, we have completely redesigned the protein database and the process that is used to generate it. As part of the reimplementation process, each non-redundant protein is now assigned a unique identifier with the prefix MGYP, instead of the sha256 digest that was previously used as an accession. Contigs are now also accessioned with the prefix MGYC. Internally, the flat files have been replaced with a MySQL database that stores information and relationships between studies, assemblies, contigs, proteins, protein metadata, and annotations (see Figure 3). The implementation of this protein and contig accessioning within the protein database represents the first step in adopting these identifiers throughout the MGnify resource, providing a reference framework for researchers in reporting and interpreting metagenomic analysis results.

These developments allow us to better address some of the requests posed by MGnify users. Specifically, common requests have been to: (i) identify the specific set of studies, assemblies and even contigs that a unique protein had been identified in; (ii) retrieve the genomic contexts for a given protein. The reimplementation of the protein database involved a programme of retrofitting older assemblies already included in previous releases, analysed using MGnify's v4.1 and v5 analysis pipelines, provision of the metadata links for this study, provision of assembly and genomic context, all while still maintaining the unique identifiers they had been assigned in previous releases. For each protein, the process populates a metadata table that stores the original contig and assembly identifiers, the protein prediction tool (Prodigal (18) or FragGeneScan (19)), whether the protein is a full length or partial sequence (based on the gene structure), and the position of the protein on the contig (start position, end position, and strand).

The current release of the MGnify protein database comprises 2 477 479 951 protein sequences. At each release this set of sequences is clustered using Linclust, part of the MMseqs2 package (20), employing coverage and identity thresholds of 0.90, resulting in a current set of 623 796 864 clusters. The clusters range in size, with the largest containing 29 209 sequences, but a substantial portion (72%, 446 078 728) are clusters of a single sequence (singletons). The clustering approach is unidirectional, meaning that similar sequences are grouped together even if one sequence is a partial prediction of another (i.e. a partial prediction from the same gene as a full length sequence would be grouped together). Notably, only 51 749 298 (12%) of the singletons are predicted to be full-length sequences, and thus singleton clusters of partial predicted protein sequences should perhaps be treated with some caution. Regardless, over 2 billion sequences are still contained within those clusters containing two or more sequences, with a mean cluster size of 11, indicating that the majority of proteins (or highly similar sequences) have been seen more than once.

In previous releases of the protein database, we also clustered the metagenomics derived sequences with UniProtKB (21,22) to calculate the overlap between the two resources. However, due to the continually increasing number of protein sequences in MGnify and the low numbers of clusters that contained a UniProt sequence, this functionality has been removed. For each cluster, we annotated the cluster representative sequences with Pfam (23) using HMMER (http://hmmer.org) with Pfam gathering thresholds (i.e. using –cut-ga parameter in HMMER), which is the curated Pfam cutoff score that represents a significant match between the Pfam model and the sequence. This provided an annotation for 285 839 621 of the 623 796 864 cluster representative sequences, indicating about half of all clusters contain some form of functional annotation.

### Extending protein annotations

As part of a collaboration with Google Research, we also provide in this release annotations produced by ProtENN2 (Bileschi *et al.*, in prep., (24)), which uses convolutional neural networks to annotate each protein residue in the database with a Pfam family (or clan) label that is then converted into domain calls. Supplementing the more classical Pfam annotations assigned by HMMER with those provided by ProtENN2 increases the overall number of sequences we can label with a functional annotation. Specifically, ProtENN2 provided 2.24 billion annotations on 1.46 billion sequences. Our estimates indicate that this reflects annotations for an additional 200 million proteins that lack any annotation using Pfam (gathering thresholds) and HMMER, which in turn provides annotations for a further 44 million cluster representatives in the protein database. Many of these ProtENN2 annotations are found to be close yet below the Pfam gathering thresholds when using HMMER, indicating that a similar signal is detected by both approaches. Given that 38.4 million cluster representatives receive an annotation solely from classical Pfam gathering thresholds, and not from ProtENN2, it is worth noting that we are not indicating that one approach is better than another, simply that the union of the annotations is more comprehensive.

### API and website improvements

**Figure 4.**
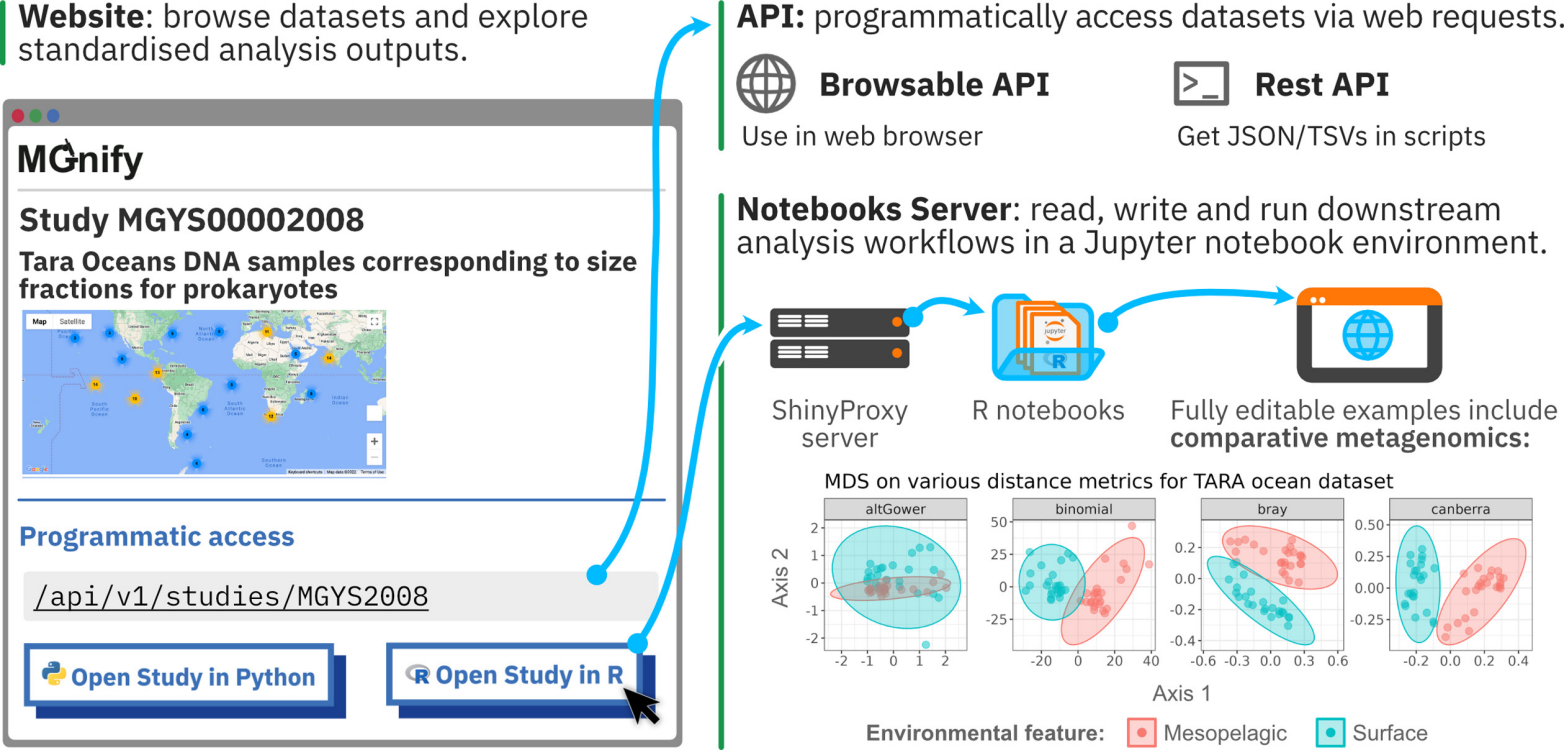
The access options for users of MGnify's web resources: website, API, and notebooks server. The redesigned website now includes links to programmatically access datasets (in this example, a study) using the API. A conceptual flow for launching an R Notebook is shown: following a deep link from the website into the notebook server, and using one of the example code notebooks. In this comparative metagenomics example available on the server, taxonomic diversity is being compared at different water depths using multidimensional scaling (MDS) and a variety of distance metrics.

MGnify's traditional web interface allows users to browse individual datasets and is complemented by an API to support the increasing demand for programmatic access to large sections of the data housed in MGnify. We have recently improved programmatic access on several fronts with a view to supporting easier access to the data for life science users (25), see Figure 4.

The MGnify API is built on top of the Django web framework. Upgrading from the Django 2 series to Django 3.2 has enabled a cascade of other updates. Notably, the translation layer between the Object Relational Mapping and the API surface is more compliant with our chosen REST-like API specification, namely JSON:API. The formal API specification is now provided according to the OpenAPI Specification (OAS) version 3, making it easier to use standard libraries to access the MGnify API. Many smaller changes to support API performance have also been made, for example, to pagination, ordering, query optimisation, and relationship rendering.

Two of MGnify's microservice APIs that provide distinct additional functionality (searching across the MAG catalogues in MGnify by sequence fragment using COBS (26) and by MAG using Sourmash (27)) are now proxied through the main MGnify API, to improve consistency and discoverability. In addition to simplifying our codebases, these microservices can now also be viewed and called via the Browsable API—the self-documenting, interactive HTML rendering of the API endpoints (https://www.ebi.ac.uk/metagenomics/api). Based on the Django Rest Framework, the Browsable API itself has been upgraded so that the filtering options for each API endpoint are rendered in the user interface. This allows users to interactively find the API URL for a query (e.g. samples only from human host-associated biomes) and copy it into a script.

Alongside these API improvements, the web client has been upgraded to modern web technologies and best practices. Together, these improvements significantly optimise the most common actions, such as browsing a large dataset, filtering it, and paging through the results.

### The MGnify Notebooks Server

To facilitate easier and wider exploration of the MGnify data than is possible via the website, we have introduced the MGnify Notebooks Server (http://notebooks.mgnify.org) as a hosted Jupyter Lab (28) environment. This environment allows users to read, write, and run code notebooks in R and Python without installing software on their own computer—the computational resources used are those of the remote host server. To demonstrate the utility of the notebooks and the API more broadly, prewritten notebooks have been made available, which are editable and interactive examples of the recommended approaches to using the MGnify API from R and Python scripts. The Notebooks Server is preinstalled with various data analysis packages, including MGnifyR (https://github.com/beadyallen/MGnifyR), a package to facilitate consumption of the MGnify API in R scripts. MGnifyR wraps the MGnify API in R functions and translates the API responses into formats familiar to the R bioinformatics ecosystem, like Phyloseq objects (29). There are example notebooks using these packages, as well as documenting their features. SIAMCAT (30) is also installed, enabling users to explore machine learning based comparative metagenomics workflows. All of the installed packages are available from public code repositories, facilitating installation in any other computing environment.

We anticipate usage of the Notebooks Server in two ways: (i) for short data retrieval and manipulation tasks like concatenating paginated data into a long TSV file, and (ii) as an interactive documentation resource for users, who can then create their own software environments and scripts on their own compute resources. Furthermore, the layers of this technology stack can also be used independently. The notebooks can be downloaded from a public GitHub repository (https://github.com/ebi-metagenomics/notebooks) and opened with any Jupyter Lab installation. The Dockerfile can be built anywhere or the image pulled from a public container repository (https://quay.io/repository/microbiome-informatics/emg-notebooks.dev). The entire stack, including ShinyProxy can be installed on any computer or suitable web server, ensuring easy and wide reuse by the community.

The Notebooks Server is integrated into the MGnify website through deep links, i.e. URLs on the website that launch an instance of the Notebooks Server in a particular state. For example, the programmatic access section of a Study page on the MGnify website reveals deep links to R and Python notebooks, with code that reads the details of that specific study from the MGnify API ready for further analysis (see Figure 4).

We intend to add further content to the Notebooks Server, including coverage of all resource types in MGnify as well as analysis workflows sourced from our user community.

## DISCUSSION

MGnify has recorded continuous growth and development since our last update. Nevertheless, there is still a gulf between the number of analysed assembled datasets in MGnify and the number of raw read (‘assemble-able’) metagenomic datasets in the ENA. As discussed previously, this can be due to multiple reasons since not all datasets are tractable for assembly, be it through lack of coverage or an inability to assemble the dataset due to memory constraints. As the number of metagenomic datasets being generated and deposited in sequence archives continues to grow at rapid speed, we are lagging behind in our attempts to assemble them. For a subset of these we have attempted but failed to generate a primary assembly. In the interests of open data and a willingness to report a negative result, we are evaluating approaches on how best to indicate when we have attempted assembly without success, the best forum to store this information, as well as to define what associated information would be useful to capture. For instance, recording the provenance of the assembly pipeline (including all versioned tools) previously tried along with the reasons for failure (e.g. maximum memory allocation exceeded) would help identify specific datasets that are tractable for future assembly attempts, provided specific improvements are carried out to the pipeline.

It is also evident from a survey of the literature that many researchers are increasingly assembling metagenomic datasets themselves. However, few of these assembled metagenomes are ever deposited (and/or appropriately labelled for discoverability) in sequence repositories. To encourage data reuse, minimisation of unnecessary compute, and establishment of data provenance, we strongly encourage researchers to submit their own primary assembled metagenomes to INSDC.

Ultimately, the ability to bridge the gap while keeping pace with the increasing volume of data being generated and submitted far exceeds our existing computational resources. Therefore, we will need to address this in the future by devising new technical solutions while also sharing this burden across the research community.

We are currently investigating the most appropriate approach to make the latest version of the protein database with its substantially increased size easily available for users. Availability in flat file format presents challenges due to the sheer volume of data. Downloading the entire database as flat files would likely be problematic for many users, let alone having access to a server capable of hosting the database. As such, we are investigating options to host the database somewhere accessible, allowing users to query it directly rather than download the content locally. In terms of further enhancements to the annotations, we plan to provide Pfam/HMMER annotations on all sequences (rather than just the cluster representatives) to complement the ProtENN2-based Pfam annotations as described above.

## DATA AVAILABILITY

MGnify services and data are freely available at (https://www.ebi.ac.uk/metagenomics/). MGnify pipelines are freely available at (https://github.com/EBI-Metagenomics). Content is distributed under the EMBL-EBI Terms of Use available at (https://www.ebi.ac.uk/about/terms-of-use), except the MGnify protein database which has been made available under a CC0 licence.

## References

[1] Lobanov V. , Gobet A., Joyce A. Ecosystem-specific microbiota and microbiome databases in the era of big data. Environ. Microbiome. 2022; 17:37. 35842686 10.1186/s40793-022-00433-1 PMC9287977

[2] Mitchell A.L. , Almeida A., Beracochea M., Boland M., Burgin J., Cochrane G., Crusoe M.R., Kale V., Potter S.C., Richardson L.J. et al. MGnify: the microbiome analysis resource in 2020. Nucleic Acids Res. 2020; 48:D570–D578. 31696235 10.1093/nar/gkz1035 PMC7145632

[3] Crusoe M.R. , Abeln S., Iosup A., Amstutz P., Chilton J., Tijanić N., Ménager H., Soiland-Reyes S., Gavrilović B., Goble C. et al. Methods included: standardizing computational reuse and portability with the common workflow language. Commun. ACM. 2022; 65:54–63.

[4] Goble C. , Soiland-Reyes S., Bacall F., Owen S., Williams A., Eguinoa I., Droesbeke B., Leo S., Pireddu L., Rodríguez-Navas L. et al. Implementing FAIR Digital Objects in the EOSC-Life Workflow Collaboratory. Zenodo. 2021; 10.5281/zenodo.4605654.

[5] Tyson G.W. , Chapman J., Hugenholtz P., Allen E.E., Ram R.J., Richardson P.M., Solovyev V.V., Rubin E.M., Rokhsar D.S., Banfield J.F. Community structure and metabolism through reconstruction of microbial genomes from the environment. Nature. 2004; 428:37–43. 14961025 10.1038/nature02340

[6] Nielsen H.B. , Almeida M., Juncker A.S., Rasmussen S., Li J., Sunagawa S., Plichta D.R., Gautier L., Pedersen A.G., Le Chatelier E. et al. Identification and assembly of genomes and genetic elements in complex metagenomic samples without using reference genomes. Nat. Biotechnol. 2014; 32:822–828. 24997787 10.1038/nbt.2939

[7] Parks D.H. , Rinke C., Chuvochina M., Chaumeil P.-A., Woodcroft B.J., Evans P.N., Hugenholtz P., Tyson G.W. Recovery of nearly 8,000 metagenome-assembled genomes substantially expands the tree of life. Nat. Microbiol. 2017; 2:1533–1542. 28894102 10.1038/s41564-017-0012-7

[8] Nayfach S. , Shi Z.J., Seshadri R., Pollard K.S., Kyrpides N.C. New insights from uncultivated genomes of the global human gut microbiome. Nature. 2019; 568:505–510. 30867587 10.1038/s41586-019-1058-x PMC6784871

[9] Almeida A. , Nayfach S., Boland M., Strozzi F., Beracochea M., Shi Z.J., Pollard K.S., Sakharova E., Parks D.H., Hugenholtz P. et al. A unified catalog of 204,938 reference genomes from the human gut microbiome. Nat. Biotechnol. 2021; 39:105–114. 32690973 10.1038/s41587-020-0603-3 PMC7801254

[10] Kanehisa M. , Sato Y., Kawashima M., Furumichi M., Tanabe M. KEGG as a reference resource for gene and protein annotation. Nucleic Acids Res. 2016; 44:D457–D462. 26476454 10.1093/nar/gkv1070 PMC4702792

[11] Richardson L.J. , Rawlings N.D., Salazar G.A., Almeida A., Haft D.R., Ducq G., Sutton G.G., Finn R.D. Genome properties in 2019: a new companion database to interpro for the inference of complete functional attributes. Nucleic Acids Res. 2019; 47:D564–D572. 30364992 10.1093/nar/gky1013 PMC6323913

[12] Blin K. , Wolf T., Chevrette M.G., Lu X., Schwalen C.J., Kautsar S.A., Suarez Duran H.G., de los Santos E.L.C., Kim H.U., Nave M. et al. antiSMASH 4.0—improvements in chemistry prediction and gene cluster boundary identification. Nucleic Acids Res. 2017; 45:W36–W41. 28460038 10.1093/nar/gkx319 PMC5570095

[13] Nassar M. , Rogers A.B., Talo’ F., Sanchez S., Shafique Z., Finn R.D., McEntyre J A machine learning framework for discovery and enrichment of metagenomics metadata from open access publications. GigaScience. 2022; 11:giac077. 35950838 10.1093/gigascience/giac077 PMC9366992

[14] Jumper J. , Evans R., Pritzel A., Green T., Figurnov M., Ronneberger O., Tunyasuvunakool K., Bates R., Žídek A., Potapenko A. et al. Highly accurate protein structure prediction with alphafold. Nature. 2021; 596:583–589. 34265844 10.1038/s41586-021-03819-2 PMC8371605

[15] Eiamthong B. , Meesawat P., Wongsatit T., Jitdee J., Sangsri R., Patchsung M., Aphicho K., Suraritdechachai S., Huguenin-Dezot N., Tang S. et al. Discovery and genetic code expansion of a polyethylene terephthalate (PET) hydrolase from the human saliva metagenome for the degradation and bio-functionalization of PET. Angew. Chem. Int. Ed Engl. 2022; 61:e202203061. 35656865 10.1002/anie.202203061 PMC7613822

[16] Inoue M. , Omae K., Nakamoto I., Kamikawa R., Yoshida T., Sako Y. Biome-specific distribution of Ni-containing carbon monoxide dehydrogenases. Extremophiles. 2022; 26:9. 35059858 10.1007/s00792-022-01259-y PMC8776680

[17] Kazlauskas D. , Krupovic M., Guglielmini J., Forterre P., Venclovas Č. Diversity and evolution of B-family DNA polymerases. Nucleic Acids Res. 2020; 48:10142–10156. 32976577 10.1093/nar/gkaa760 PMC7544198

[18] Hyatt D. , Chen G.-L., Locascio P.F., Land M.L., Larimer F.W., Hauser L.J. Prodigal: prokaryotic gene recognition and translation initiation site identification. BMC Bioinformatics. 2010; 11:119. 20211023 10.1186/1471-2105-11-119 PMC2848648

[19] Rho M. , Tang H., Ye Y. FragGeneScan: predicting genes in short and error-prone reads. Nucleic Acids Res. 2010; 38:e191. 20805240 10.1093/nar/gkq747 PMC2978382

[20] Steinegger M. , Söding J. Clustering huge protein sequence sets in linear time. Nat. Commun. 2018; 9:2542. 29959318 10.1038/s41467-018-04964-5 PMC6026198

[21] UniProt Consortium UniProt: a worldwide hub of protein knowledge. Nucleic Acids Res. 2019; 47:D506–D515. 30395287 10.1093/nar/gky1049 PMC6323992

[22] Boeckmann B. , Bairoch A., Apweiler R., Blatter M.-C., Estreicher A., Gasteiger E., Martin M.J., Michoud K., O’Donovan C., Phan I. et al. The SWISS-PROT protein knowledgebase and its supplement TrEMBL in 2003. Nucleic Acids Res. 2003; 31:365–370. 12520024 10.1093/nar/gkg095 PMC165542

[23] Mistry J. , Chuguransky S., Williams L., Qureshi M., Salazar G.A., Sonnhammer E.L.L., Tosatto S.C.E., Paladin L., Raj S., Richardson L.J. et al. Pfam: the protein families database in 2021. Nucleic Acids Res. 2021; 49:D412–D419. 33125078 10.1093/nar/gkaa913 PMC7779014

[24] Bileschi M.L. , Belanger D., Bryant D.H., Sanderson T., Carter B., Sculley D., Bateman A., DePristo M.A., Colwell L.J. Using deep learning to annotate the protein universe. Nat. Biotechnol. 2022; 40:932–937. 35190689 10.1038/s41587-021-01179-w

[25] Tarkowska A. , Carvalho-Silva D., Cook C.E., Turner E., Finn R.D., Yates A.D. Eleven quick tips to build a usable REST API for life sciences. PLoS Comput. Biol. 2018; 14:e1006542. 30543619 10.1371/journal.pcbi.1006542 PMC6292566

[26] Bingmann T. , Bradley P., Gauger F., Iqbal Z. COBS: a compact bit-sliced signature index. String Processing and Information Retrieval. 2019; Cham Springer International Publishing 285–303.Lecture notes in computer science.

[27] Titus Brown C. , Irber L. sourmash: a library for minhash sketching of DNA. J. Open Source Softw. 2016; 1:27.

[28] Kluyver T. , Ragan-Kelley B., Pérez F., Granger B., Bussonnier M., Frederic J., Kelley K., Hamrick J., Grout J., Corlay S. et al. Loizides F. , Schmidt B. Jupyter Notebooks – a publishing format for reproducible computational workflows. 2016; 87–90.

[29] McMurdie P.J. , Holmes S. phyloseq: an r package for reproducible interactive analysis and graphics of microbiome census data. PLoS One. 2013; 8:e61217. 23630581 10.1371/journal.pone.0061217 PMC3632530

[30] Wirbel J. , Zych K., Essex M., Karcher N., Kartal E., Salazar G., Bork P., Sunagawa S., Zeller G. Microbiome meta-analysis and cross-disease comparison enabled by the SIAMCAT machine learning toolbox. Genome. Biol. 2021; 22:93. 33785070 10.1186/s13059-021-02306-1 PMC8008609

